# Prognostic Autophagy-Related Genes of Gastric Cancer Patients on Chemotherapy

**DOI:** 10.3389/fgene.2021.720849

**Published:** 2021-10-25

**Authors:** Xiaolong Liu, Bin Ma, Mali Chen, Yaqing Zhang, Zhen Ma, Hao Chen

**Affiliations:** ^1^ NHC Key Laboratory of Diagnosis and Therapy of Gastrointestinal Tumor, Gansu Provincial Hospital, Lanzhou, China; ^2^ Department of Surgical Oncology, Lanzhou University Second Hospital, Lanzhou, China; ^3^ Department of Obstetrics, Gansu Province Maternity and Child-Care Hospital, Lanzhou, China; ^4^ Department of Gynaecology, Gansu Province Maternity and Child-Care Hospital, Lanzhou, China

**Keywords:** gastric cancer, autophagy-related genes, chemotherapy, CXCR4, prognosis

## Abstract

**Background:** Chemotherapy resistance based on fluorouracil and cisplatin is one of the most encountered postoperative clinical problems in patients diagnosed with gastric cancer (GC), resulting in poor prognosis.

**Aim of the Study:** This study aimed to combine autophagy-related genes (ARGs) to investigate the susceptibility patients with GC to postoperative chemotherapy.

**Methods:** Based on The Cancer Genome Atlas (TCGA) database, gene expression data for GC patients undergoing chemotherapy were integrated and analyzed. Prognostic genes were screened based on univariate and multivariate analysis regression analysis. Subjects were divided into high-risk and low-risk groups according to the median risk score. Kaplan-Meier method was used to evaluate OS and DFS. The accuracy of the prediction was determined by the subject operating characteristic curve analysis. In addition, stratified analyses based on different clinical variables was performed to assess the correlation between risk scores and clinical variables. Quantitative real-time (qRT) PCR was used to verify the expression of CXCR4 in GC tissues and cell lines.

**Results:** A total of nine ARGs related to the prognosis of chemotherapy patients were screened out. Compared with normal gastric mucosa cell, CXCR4 showed elevated expression in GC and was significantly associated with survival. Based on GEO and TCGA databases, the model accurately predicted DFS and OS after chemotherapy.

**Conclusion:** This study established prognostic markers based on nine genes, predicting that ARGs are related to chemotherapy susceptibility of GC patients, which can provide better individualized treatment regimens for clinical practice.

## Introduction

Gastric cancer (GC) is a major health problem worldwide, which is also a challenge resulted in huge economical burdens. In East Asian countries, especially in China, GC has the highest incidence and mortality rates (GBD 2017 Stomach Cancer Collaborators, 2020). Although overall survival has improved over the past few decades, the prognosis still remains remarkedly poor (Boya et al., 2013a). Drug resistance of chemotherapeutic drugs is the main factor that causes a poor prognosis in GC patients. Conventional evaluation indexes cannot appropriately evaluate the prognosis of patients with chemotherapy, so it is necessary to have some explicit knowledge and explore victims undergoing chemotherapy.

Autophagy is an important process of eukaryotic transformation of intracellular structures and components (Boya et al., 2013b). In this process, cells wrap their own cytoplasmic proteins or organelles through a single or double membrane to form autophagosomes, which further fuse with lysosomes to form autolysosome, and degrade the contents of the package. According to the different ways of transporting cellular material to lysosomes, autophagy is divided into three types, namely, Macro-autophagy, Micro-autophagy and Chaperon mediated autophagy (CMA) (Kaushik and Cuervo, 2018). What we usually refer to as autophagy is Macro-autophagy. Unlike Macro-autophagy, there is no process of autophagosome formation in Micro-autophagy. The lysosomal membrane itself invades, wrapping and phagocytosing the material to be degraded in the cell, and degrading it. Unlike the former two, CMA is selective in protein degradation. The protein in the cell is restored from the folded state to the unfolded state, and then transferred to the lysosome (Tekirdag and Cuervo, 2018).

Physiological imbalance problems in some processes of autophagy can lead to various diseases and ailments, such as cancer (Levine and Kroemer, 2019). There are some significant pathophysiological processes with autophagy regard to some malignancies (Shen et al., 2008). For instance, Beclin1 gene is associated with autophagy to some extent, which is highly expressed in GC, but not or low expressed in normal tissues (Qu et al., 2017). Glutamine decomposition provides energy for tumor cells, and autophagy activation also contribute to abnormal glutamine decomposition in GC cells, promoting promotion and metastasis (Zhang et al., 2018). LC3 has been widely used as a biomarker for autophagosome, with high expression of LC3 detected in 58% of GC cells, but not in normal gastric epithelial cells (Yoshioka et al., 2008). P62/SQSTM1, a characteristic substrate of ubiquitin-protein in autophagy, which is more significantly up-regulated in GC specimens than in normal gastric mucosa (Kim et al., 2019), while the interpretation of P62/SQSTM1 has some adverse clinical outcomes of the ailment (Masuda et al., 2016). However, whether these autophagy-related genes (ARGs) are correlated with GC patient prognosis remains highly unknown.

Chemotherapy, remains the standard treatment against advanced GC, can exert cytotoxic *via* inducing and enhancing autophagy. It has been reported that autophagy is a survival mechanism that contributes to the development of acquired drug resistance. For instance, autophagy can inhibit the apoptosis of 5-FU-induced MGC803 in GC cells (Korourian et al., 2019). Aquaporin 3(AQP3) promotes the resistance of GC cells to cisplatin *via* autophagy (Dong et al., 2016). Consequently, autophagy might have a fundamental impact on the chemotherapy response of GC. Therefore, it is important to analyze the expression patterns of ARGs in the GC patients on chemotherapy, as well as their prognostic value.

On this basis, our study used bioinformatics methods to predict the prognosis of chemotherapy in GC patients by screening ARGs. This model is helpful for clinicians to develop more individualized chemotherapy regimens and serve patients better and more efficiently. The expression of CXCR4 were verified in GC tissues and cells by qRT.

## Materials and Methods

### Data Collection

ARGs were downloaded and organized from the Human Autophagy Databases (http://autophagy.lu/clustering/index.html). Chemotherapy regimens based on cisplatin and fluorouracil were widely used. Therefore, gene expression data and clinical information were obtained from The Cancer Genome Atlas (TCGA) data portal (https://portal.gdc.cancer.gov/) in 157 patients with GC who received cisplatin or fluorouracil post operatively. The GSE26253 gene expression profile with 432 patients on chemotherapy was downloaded from the Gene Expression Omnibus (GEO) database.

### Differential Expression of ARGs and the Enrichment Analysis

The differentially expressed genes (DEGs) of ARGs between chemotherapy group and adjacent nontumorous samples were identified using “limma” R package with a false discovery rate (FDR) <0.05 in the TCGA cohort. To explore the main biological characteristics of ARGs related to chemotherapy, Gene Ontology (GO) and Kyoto Encyclopedia of Genes and Genomes (KEGG) analysis were performed by the “clusterProfiler” R package.

### Construction of Prognostic Gene Signatures

To identify the prognostic value of ARGs with overall survival (OS) and disease-free survival (DFS) in GC chemotherapy group, univariate Cox proportional hazard regression analysis was performed based on TCGA and GEO database. The prognostic model of ARGs was established by multivariate Cox regression analysis. The risk score was calculated based on the expression level of ARGs. Optimal cutoff values were used to divide patients into low-risk and high-risk groups. In addition, Kaplan-Meier method was used to conduct survival analysis based on risk score. To investigate whether the ARGs risk index in the TCGA cohort could be an independent predictor of OS, univariate and multivariate Cox regression analyses were further applied. Risk score, age, sex, tumor subtype, pathological stages, and histological grades were used as covariates. The correlation between risk score and clinicopathological variables was calculated by using the T-test. *p* < 0.05 was considered statistically significant. The Kaplan-Meier plotter database was constructed based on gene chips and RNA-seq data from public databases such as GEO, EGA, and TCGA. We used the Kaplan-Meier plotter database to analyze the relationship between the expression of CXCR4 and the prognosis of GC, we selected “Pan-cancer RNA-seq” and “Stomach adenocarcinoma.”

### Gene Set Enrichment Analysis (GSEA)

GSEA was conducted to explore the characteristics of gene Hallmarks in high-risk and low-risk populations. GSEA was performed using GSEA3.0 (http://www.broad.mit.edu/gsea/). Differences for which the nominal *p* < 0.05 and the FDR < 0.25 were considered statistically enriched.

### Tissue Samples

A total of 60 GC cancerous and paracancerous tissue samples were collected in the surgery from May 2010 to December 2018, and the tissues were stored at a −80°C freezer. All patients enrolled in the study signed written informed consent. None of the subjects underwent radiotherapy or chemotherapy prior to the surgery. The tissues were subjected to homogenization, and then total RNA was extracted for RT-PCR. The study was approved by the Clinical Research Ethics Committees.

### Cell Culture

Human GC cell lines MKN45, AGS, HGC27, N87 and human normal gastric mucosal epithelial cells GES-1 were purchased from Cell Culture Center of Chinese Academy of Medical Sciences (Beijing, China). All cells were cultured in RPMI 1640 medium supplemented with 10% fetal bovine serum (Gibico, United States) and 1% penicillin and streptomycin (Biyuntian, China).

### Quantitative Real-Time (qRT) PCR

According to the manufacturer’s instructions, the total RNA of the cells was extracted using Trizol reagent (Accurate Biology, China). β-actin as endogenous control, the relative expression of target gene was detected by SYBR Green method on Bio-Rad CFX96GRT-PCR system. The primer sequences were as follows (“F” represents “forward”; “R” represents “reverse”). CXCR4,5′-GGCCCTCAAGACCACAGTC-3′(F), 5′-TTAGCTGGAGTGAAAACTTG-3′(R). Relative quantification of mRNA expression was calculated using the 2^−ΔΔct^ method (Livak and Schmittgen, 2001).

### Statistical Analysis

Student’s t-test was used to compare gene expression between tumor GC on chemotherapy and normal tissues. Univariate and multivariate cox regression analyses were used to identify independent factors of OS and DFS. Kaplan–Meier curve was implemented to visualize the survival. R software (version 4.0.2) was applied to process and analyze the statistics.

## Results

### Identification of the Differentially Expressed ARGs in TCGA Cohort

**FIGURE 1:**
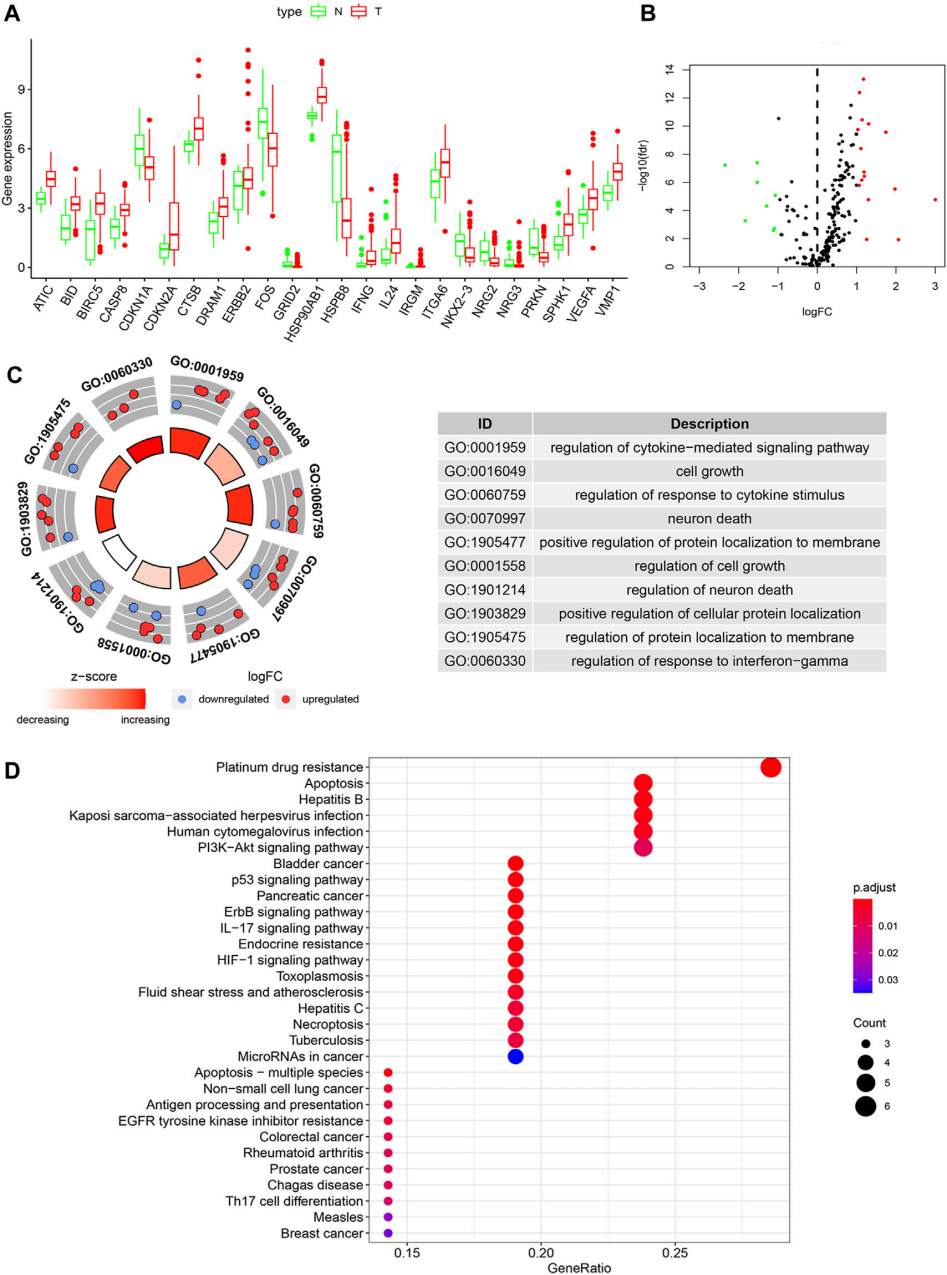
The differentially expressed ARGs and function analysis in a chemotherapeutic group and normal tissues. **(A)** Visualization of the expression levels of the 24 differentially expressed ARGs. **(B)** Volcano plot of 221 ARGs. Red upregulation; Green downregulation. **(C)** GO analysis of 24 differentially expressed ARGs. **(D)** Bubble diagram of KEGG enrichment analysis.

232 ARGs were obtained in our study. A total of 221 ARGs were expressed in TCGA cohort. The results were 157 patients who received chemotherapy and 32 normal samples. The correlated basic clinical characteristics was also compared, as shown in Table 1. With FDR < 0.05 and |log2 FC| > 1 as the screening criteria, 24 DEGs of ARGs were identified (Figures 1A,B). The upregulated ATGs were IFNG, ATIC, BIRC5, CASP8, VMP1, IL24, CDKN2A, HSP90AB1, VEGFA, CTSB, and ERBB2. The downregulated ATGs include: PRKN, CDKN1A, GRID2, HSPB8, NRG3, NRG2, FOS, and NKX2-3.

**TABLE 1:** Clinical characteristics of GC patients with chemotherapy in TCGA cohort.

Characteristic	Variables	Total	Percentage (%)
Age	≤65	184	44.9
	>65	226	55.1
Sex	Male	263	64.1
	Female	147	35.9
Grade	G1-2	151	36.8
	G3	251	61.2
	GX	8	2.0
Lauren classification	Intestinal	182	44.4
	Diffuse	74	18.0
	Mixed	154	37.6
Stage	I	57	13.9
	II	129	31.5
	III	181	44.1
	IV	43	10.5
T stage	T1	19	4.6
	T2	84	20.5
	T3	191	46.6
	T4	116	28.3
N stage	N0	130	31.7
	N1	107	26.1
	N2	83	20.2
	N3	90	22.0
M stage	M0	363	88.5
	M1	29	7.1
	Mx	18	4.4

### Enrichment Analysis of the Differentially Expressed ARGs

We utilized GO enrichment and KEGG pathway analysis to explore the possible biological functions in GC that may be associated with chemotherapy response. Based on GO analysis, the differentially expressed ARGs were mainly enriched in cell growth, neuron death, positive regulation of protein localization to membrane, autophagy (Figure 1C). The KEGG pathways analysis indicated that the DEGs were mainly related to platinum drug resistance, apoptosis, EGFR tyrosine kinase inhibitor resistance and p53 signaling pathway (Figure 1D).

### The Construction of Prognostic Markers of ARGs for OS in TCGA Cohort

**FIGURE 2:**
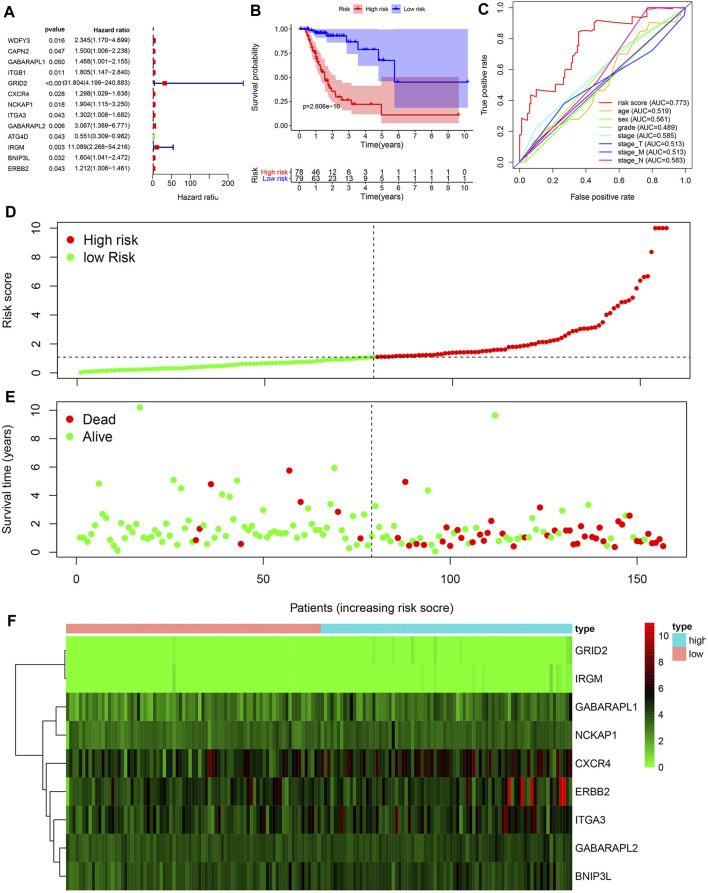
Construction of a prognosis-related risk signature. **(A)** Univariate Cox regression analysis of ARGs related to overall survival of GC patients with chemotherapy. **(B)** Kaplan-Meier OS curves for TCGA GC patients treated with chemotherapy by median risk. **(C)** Multi-index ROC curve of risk score and other indicators. **(D)** Distribution of the risk scores of GC patients. **(E)** The number of survivors and non-survivors with different risk scores. **(F)** The expression of nine ARGs in the **high**- and **low**-risk groups.

Then 221 ATGs were analyzed by univariate Cox regression analysis. Thirteen ARGs were associated with the prognostic of patients with chemotherapy in TCGA cohort (Figure 2A). After multivariate Cox regression analysis, nine ARGs were finally identified to relate to the OS. The coefficients of each gene were shown in Table 2.

**TABLE 2:** Multivariate Cox regression analysis of prognostic genes.

Gene	Co-ef	HR	HR.95L	HR.95H
GABARAPL1	0.370661	1.448692	0.912786	2.299233
GRID2	2.358799	10.57824	0.898029	124.6053
CXCR4	0.302963	1.353864	1.034964	1.771025
NCKAP1	0.71455	2.043268	0.967303	4.316067
ITGA3	0.269185	1.308897	0.971892	1.762759
GABARAPL2	1.334027	3.796301	1.55472	9.26977
IRGM	2.963281	19.36138	1.362477	275.1335
BNIP3L	0.592749	1.808954	1.091792	2.997195
ERBB2	0.319098	1.375887	1.105664	1.712152

### ARGs as an Independent Prognostic Factor for OS of GC Patients in Chemotherapy Group

Risk scores were calculated based on ATGs mRNA expression levels and risk factors. Patients were divided into high-risk and low-risk groups according to the median risk score. Kaplan-Meier analysis demonstrated that high risk score was associated with poor prognosis, and the 5-year survival rates were 16.5 and 7.7%, respectively, in the high and low risk groups (Figure 2B). ROC curves were constructed to determine the ability of ARGs prediction for patients in chemotherapy group (Figure 2C). The area under the curve (AUC) of the ARGs for OS was 0.773, which was significantly higher than other indicators. The risk scores of the high-risk and low-risk groups were visualized (Figure 2D). As the risk score increased, the number of deaths increased (Figure 2E). Heatmaps were constructed for both groups (Figure 2F). These results suggested that risk scores accurately reflected patient survival.

**FIGURE 3:**
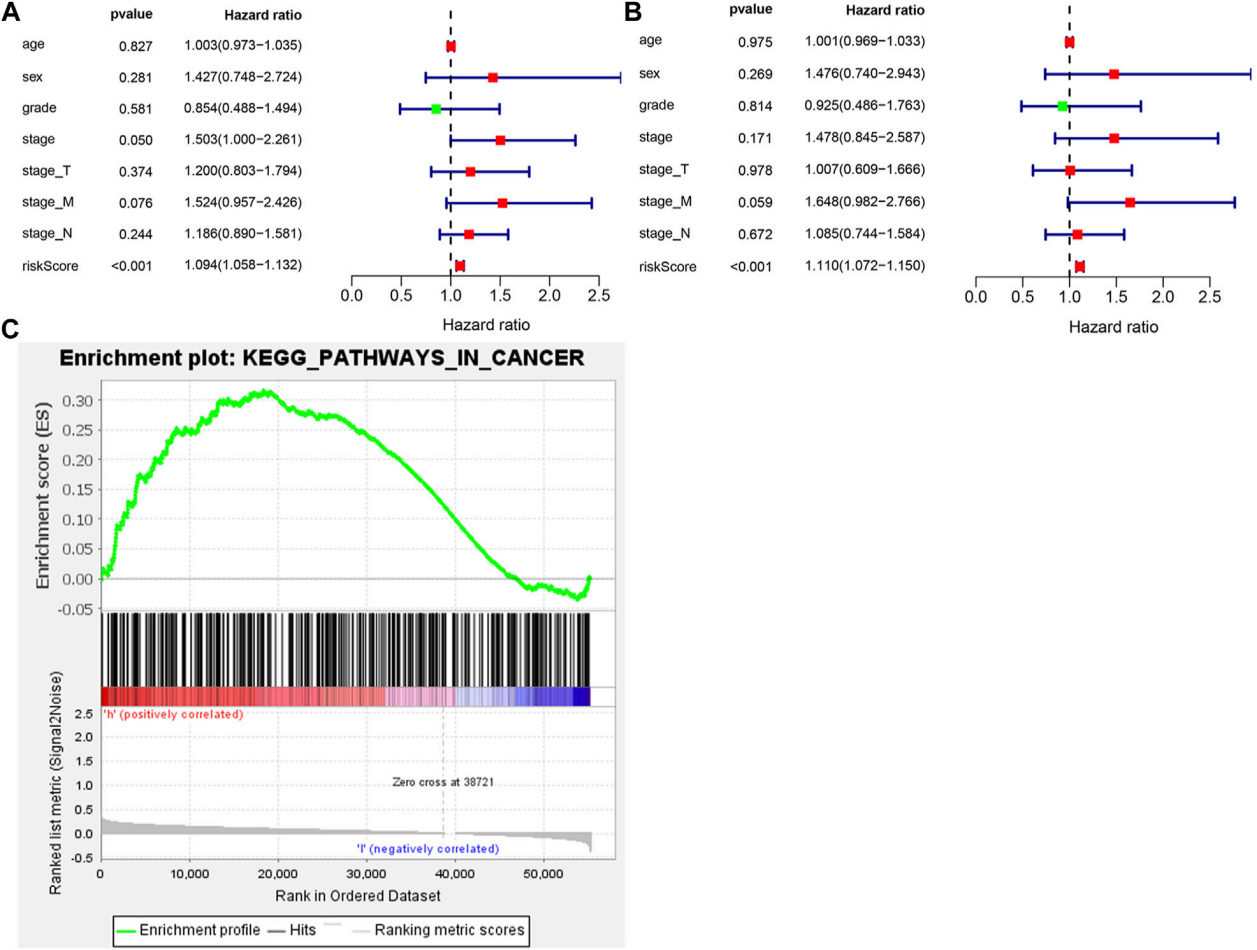
The ATGs for OS is an independent prognostic factor for GC. **(A)** Univariate Cox regression analysis of correlations between the risk score for OS and clinical variables. **(B)** Multivariate Cox regression analysis of correlations between the risk score for OS and clinical variables. **(C)** Gene set enrichment analysis comparing the **high**- and **low**-risk groups.

To determine whether autophagy-related scoring features were independent prognostic factors in GC patients undergoing chemotherapy, Cox regression analysis was performed. Similarly, the significant correlation between risk scores and OS was achieved by the univariate Cox regression analysis (HR = 1.094, 95%CI = 1.058–1.132, *p* < 0.001) (Figure 3A). Multivariate Cox regression analysis showed that risk score was an independent factor affecting the prognosis of CG patients undergoing chemotherapy (HR = 1.110, 95%CI = 1.072–1.150, *p* < 0.001) (Figure 3B). Considering the survival differences between the high-risk and low-risk groups, we conducted GSEA to investigate the functional differences between the two groups (Figure 3C). Cancer pathways were enriched, suggesting that autophagy is involved in the regulation of chemotherapy for high-risk GC patients. Furthermore, the expression of CXCR4 in GC cell lines was detected by qRT PCR. Compared with normal gastric mucosa epithelial cells, the expression of CXCR4 was significantly increased in GC cell lines.

### Relationship Between the Prognostic ARGs for OS and Clinicopathological Variables

**FIGURE 4:**
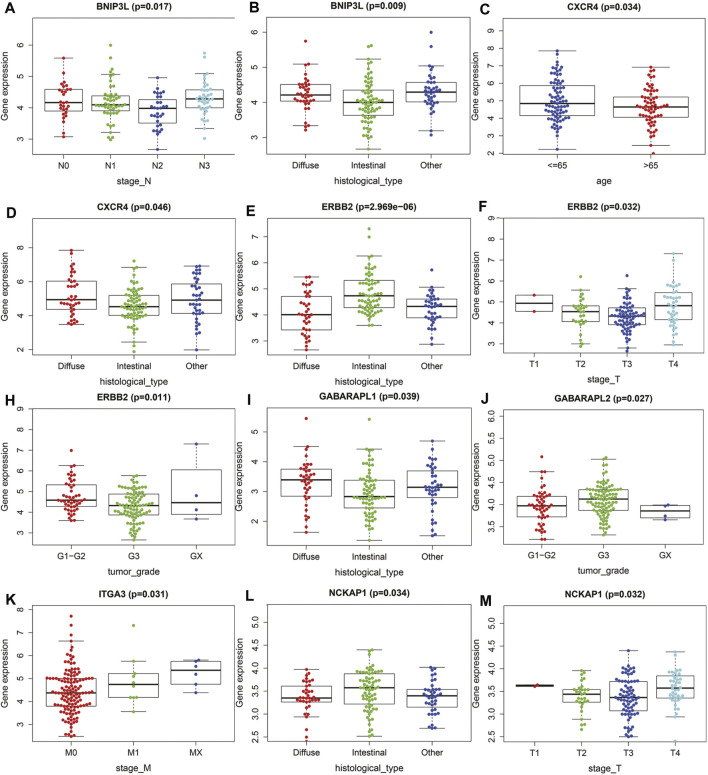
The relationships between the ATGs and clinicopathological variables. **(A,B)** BNIP3L. **(C,D)** CXCR4. **(E,F,H)** ERBB2. **(I,J)** GABRAPL. **(K)** ITGA3. **(L,M)** NCKAP1.

To determine whether ARGs affect the progression of gastric cancer, we analyzed the correlation between OS autophagy-related genes and clinicopathological variables. Figure 4 showed that BNIP3L, CXCR4, ERBB2, GABRAPL, ITGA3, and NCKAP1 were significantly correlated with the pathological classification of GC. On the one hand, BNIP3L, CXCR4, ERBB2, GABRAPL, and NCKAP1 were significantly correlated with Lauren typing. ERBB2 and GABRAPL were also significantly correlated with tumor grade. BNIP3L, ERBB2, ITGA3, and NCKAP1 were significantly correlated with TNM staging.

### Prognostic ARGs for DFS of GC Patients in Chemotherapy Group

**FIGURE 5:**
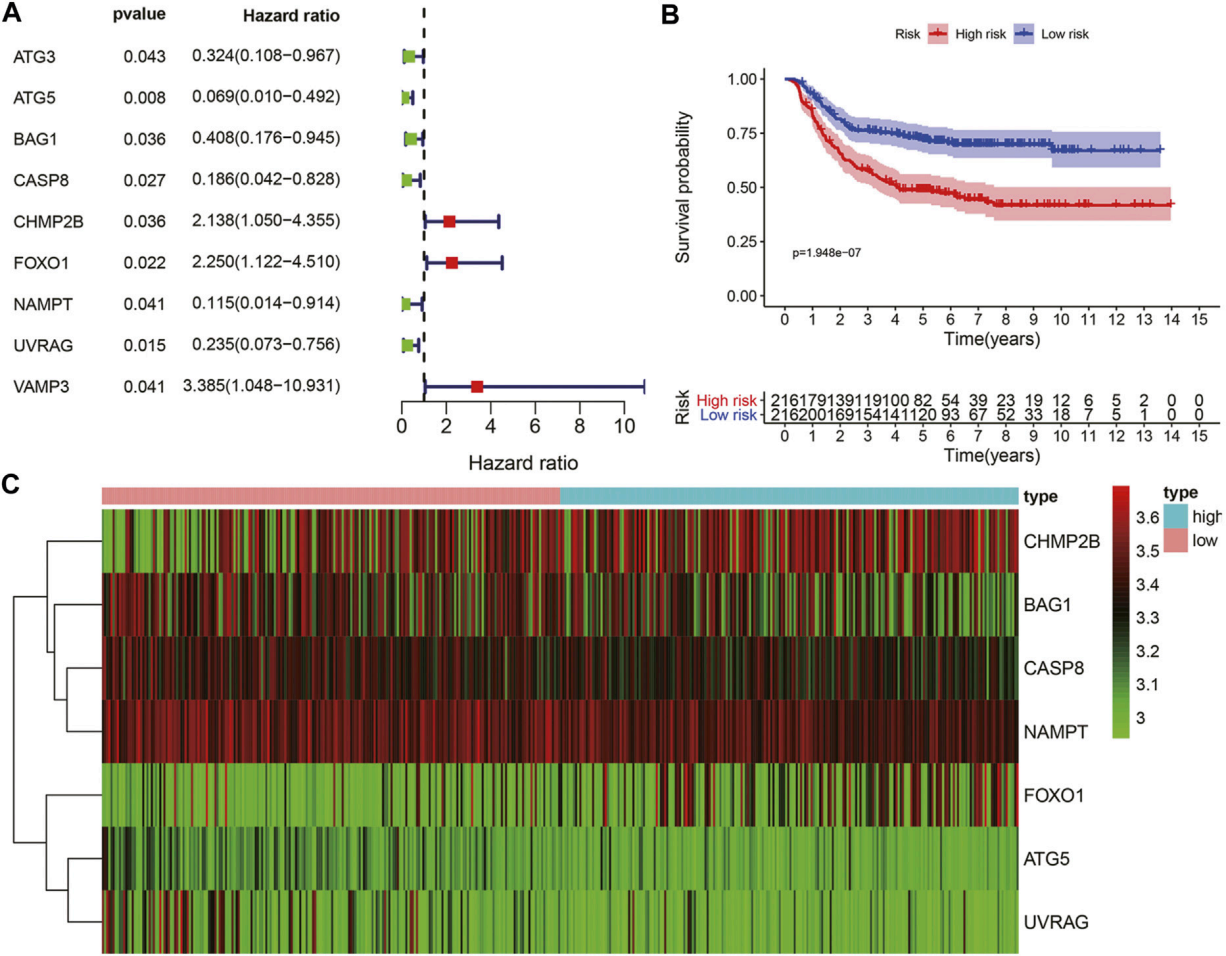
The ATGs for DFS is an independent prognostic factor for GC. **(A)** Univariate Cox regression analysis of autophagy genes related to DFS of GC patients with chemotherapy. **(B)** Kaplan-Meier DFS curves for **high** and **low**-risk groups. **(C)** The expression of seven autophagy-related genes in the **high** and **low**-risk groups.

Considering the significance of DFS in the prognosis of GC patients undergoing chemotherapy, we also established a prognostic marker for DFS. GSE26253 dataset was incorporated. According to univariate Cox regression analysis, there was a certain significant correlation among the nine ARGs (Figure 5A). After multivariate Cox regression analysis, we finally obtained seven ARGs and divided the patients in the whole data set into high-risk group and low-risk group according to the median of risk scores. Kaplan-Meier analysis revealed that the DFS in the high-risk group was significantly shorter than that in the low-risk group (*p* < 0.001, Figure 5B). Heatmaps were developed for both groups (Figure 5C). These results suggest that the prognostic marker of DFS can also predict the prognosis of GC patients undergoing chemotherapy.

### Validation of CXCR4 in Independent GC Cohorts

**FIGURE 6:**
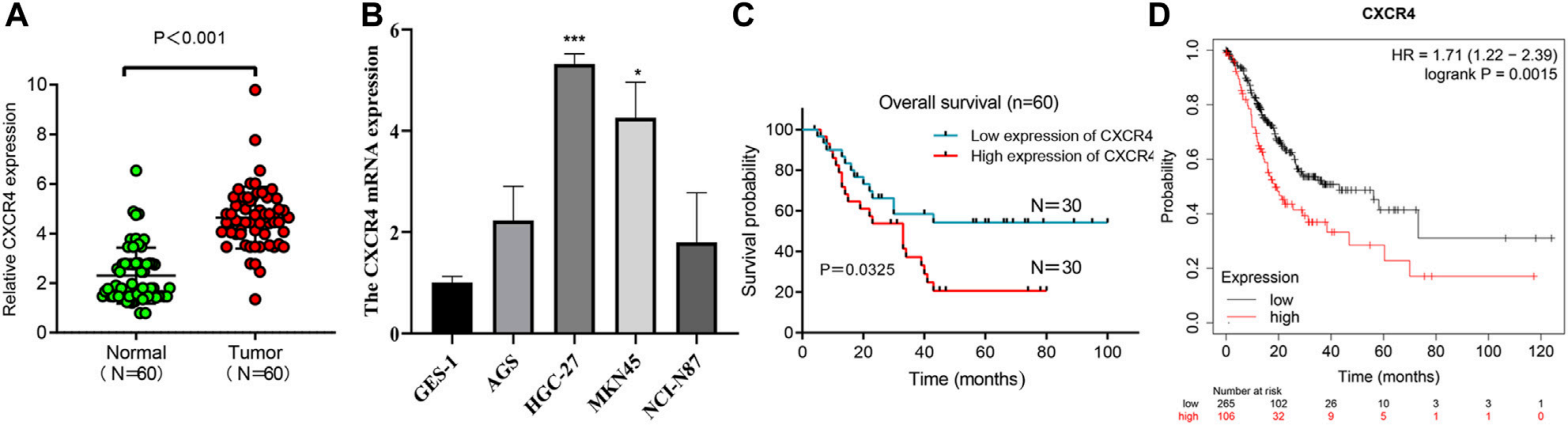
CXCR4 expression in GC. **(A)** The expression of CXCR4 in GC tissues and paracancerous tissues. **(B)** CXCR4 expression in GC cell lines. **(C)** Survival analysis of CXCR4 in GC patients. **(D)** survival curve of CXCR4 based on Kaplan Meier Plotter.

We used qRT-PCR to characterize the expression of CXCR4 in GC tissues and paracancerous tissues. CXCR4 expression in GC tissues was observably higher than that in paracancerous tissues (Figure 6A). The expression of CXCR4 was elevated in GC cell lines (Figure 6B). Then, we explored the effects of CXCR4 on prognosis of GC patients and found that higher expression of CXCR4 was significantly associated with poor survival (Figure 6C). The survival curve based on Kaplan Meier Plotter showed that high CXCR4 expression was closely associated with poor prognosis in GC patients (Figure 6D).

## Discussion

The treatment cost of advanced gastric cancer is very high, and the prognosis is quite poor, so it has caused huge economic challenges on a global scale. The drug resistance of gastric cancer patients is the main reason for this phenomenon (Tsai et al., 2020). Autophagy is a process by which the body itself regulates cellular mechanisms and homeostasis (Hou et al., 2020). Some recent studies have shown that autophagy may be closely related to the resistance of chemotherapy drugs in GC patients (Peng et al., 2020; Zhao et al., 2020). Studies have found that the autophagy of GC cells with enhanced chemotherapy-drug resistance, and inhibition of autophagy can eliminate chemotherapy-resistance (Xu et al., 2018; Guo et al., 2019). Considering the importance of autophagy in chemotherapy resistance of GC, we further explored the prognostic value of autophagy in the treatment of GC. In recent years, with the advancement of genome sequencing, biochips, and high-throughput sequencing technologies, more and more studies have applied bioinformatics methods to the analysis of chip data sets, providing an effective method for the diagnosis, treatment and prognosis of gastric cancer. In this study, we combined TCGA and GEO databases to accomplish our work. The prognosis of GC patients receiving postoperative chemotherapy was analyzed. We also studied the biological function and role of ARGs in GC.

First and foremost, the differentially expressed ARGs between GC chemotherapy group and normal stomach were identified in our study. Furthermore, GO and KEGG analysis showed that the differentially expressed ARGs were mainly enriched in platinum resistance (Wu et al., 2018; Su et al., 2019; Herhaus et al., 2020). Research has demonstrated that a combination of inhibitors in GC can improve cisplatin resistance, which is consistent and concurs with our results. ARGs can promote progress in GC disease progress through platinum resistance. Moreover, there were 13 genes associated with prognosis in the GC chemotherapy group. We used multivariate Cox regression to construct and compute data set for nine genes.

Among the nine genes in the prognostic model that we constructed, GABARAPL1 knockdown has been shown to inhibit the growth of prostate cancer cells *in vitro* or *in vivo* (Keulers et al., 2015). In head and neck squamous cell carcinoma, the high expression level of GABARAPL1 is associated with the poor prognosis of patients (Liu et al., 2014). However, in certain cancers, high levels of GABARAPL1 expression are associated with better results, such as hepatocellular carcinoma (HCC) (Berthier et al., 2010) and node-positive breast cancer (Zhang et al., 2018). GC cells activate autophagy through GABARAPL1 to supplement glutamine breakdown and promote the growth and metastasis of GC cells (Ali et al., 2017). The GluD2 protein encoded by GRID2 is a member of the ionotropic glutamate receptor family that mediates excitatory synaptic transmission (Ngollo et al., 2017). Ngollo et al. showed that GRID2 is significantly overexpressed in prostate cancer (Zhong et al., 2019). NCKAP1 is abnormally expressed in HCC and used as an independent prognostic factor for patients (Teng et al., 2016). High expression level of NCKAP1 is associated with poorer survival in breast cancer patients (Jiao et al., 2019). ITGA3 has been confirmed to be associated with poor prognosis in a variety of cancers (Miao et al., 2010; Wang et al., 2019; Tian et al., 2020). Miao et al. found that GABARAP is overexpressed in colorectal cancer, and patients with high GABARAP expression have a shorter survival time (Song et al., 2015). IRGM has been shown to be dysregulated in GC and affect the occurrence and development of GC (Burada et al., 2012). BNIP3L has different expressions in a variety of cancers. It is highly expressed in HCC (Chen et al., 2020), breast cancer (Real et al., 2005) and ovarian cancer (Jia et al., 2020). However, in colorectal and pancreatic cancers BNIP3 is frequently epigenetically silenced (Mellor and Harris, 2007).

CXCR4 is a chemokine receptor, which is highly expressed in breast cancer patients, and high expression indicates a poor prognosis. We also got the same result in ovarian cancer (Jiang et al., 2006; Mirisola et al., 2009). These findings suggest that CXCR4 is a promising prognostic factor. In addition, CXCR4 also plays an important role in the chemotherapy resistance of a variety of malignant tumors. Gemcitabine is a chemotherapeutic agent for the treatment of advanced and metastatic pancreatic cancer. However, chemotherapy resistance is a critical factor affecting the clinical prognosis of pancreatic cancer. Studies have shown that activation of Akt and ERK signaling pathways mediate the resistance of pancreatic cancer to gemcitabine. Blocking CXCR4 can effectively eliminate these survival signals and restore the sensitivity of pancreatic cancer cells to gemcitabine (Singh et al., 2010). Another study confirmed that targeting CXCR4 can inhibit the growth of pancreatic cancer cells and increase the sensitivity of pancreatic cancer cells to gemcitabine (Khan et al., 2020). Similar results have been observed in colorectal cancer, miR-193a-5p reduces the chemotherapy resistance of colorectal cancer to 5-FU and oxaliplatin by targeting CXCR4 (Azar et al., 2021). An analysis based on clinical samples showed that ovarian cancer patients with high expression of CXCR4 were significantly less sensitive to chemotherapy and had a poor prognosis, suggesting that CXCR4 is the key molecules for chemotherapy resistance (Li et al., 2014). In acute myeloid leukemia, targeting CXCR4 has been proven to be one of the potential treatment methods to overcome chemotherapy resistance (Cho et al., 2015). In view of the correlation between CXCR4 and chemotherapy resistance of various tumors, we investigated the role of CXCR4 gene in GC. We have verified the expression of CXCR4 in gastric cancer cells. Our results show that the expression of gastric cancer cells is higher than that of normal gastric epithelial cells, and the expression level of gastric cancer tissues is also high. Beside the cancer, the prognosis of patients with high expression of CXCR4 is also worse than that of patients with low expression of CXCR4. Our results indicate that CXCR4 can be used as a prognostic indicator for patients with gastric cancer. GSEA results showed that autophagy regulation was mainly concentrated in the high-risk group, suggesting that autophagy in the high-risk group may regulate the tolerance of GC patients to chemotherapy and thus lead to poor prognosis (Samiei et al., 2020; Zhang et al., 2020). This is consistent with previous research.

## Conclusion

In conclusion, we constructed autophagy related genes for OS and DFS in patients with GC undergoing chemotherapy. It may perovide alternative choices for treatment strategies of GC patients with chemotherapy resistant. At the same time, CXCR4 may be used as a promising prognostic indicator for gastric cancer. However, our study still has considerable limitations. First, due to insufficient data, it is not possible to evaluate the prognostic capacity of autophagy related genes in other independent GC data sets, only a strong prospective cohort can actually evaluate predictability of the provided prognostic markers accurately. In addition, there are other prognostic factors that affect patients receiving chemotherapy, such as tumor immune microenvironment, which requires further research. Secondly, further *in vivo* and *in vitro* experiments need to be carried out to explore the expression of genes other than CXCR4 in GC tissues and the potential mechanism. Although previous studies have constructed GC-related prognostic models based on ARGs, there is no model to evaluate the prognosis of patients receiving chemotherapy.

## Data Availability

The datasets presented in this study can be found in online repositories. The names of the repository/repositories and accession number(s) can be found in the article/supplementary material.
